# Acknowledgement to Reviewers of *Toxins* in 2017

**DOI:** 10.3390/toxins10010037

**Published:** 2018-01-09

**Authors:** Toxins Editorial Office

**Affiliations:** MDPI AG, St. Alban-Anlage 66, 4052 Basel, Switzerland; toxins@mdpi.com

Peer review is an essential part in the publication process, ensuring that *Toxins* maintains high quality standards for its published papers. In 2017, a total of 400 papers were published in the journal. Thanks to the cooperation of our reviewers, the median time to first decision was 17 days and the median time to publication was 42 days. The editors would like to express their sincere gratitude to the following reviewers for their time and dedication in 2017:
Abeni, FabioGuo, FenghaiPerrone, GiancarloAbou-Mansour, ElianeGutierrez, SantiagoPetitpas, Christian M.Abraham, AnnGutiérrez, JoséPetrie-Hanson, LoraAbrunhosa, LuisGuzmán-Guillén, R.Pezzolesi, LauraAccardi, RositaHaesaert, GeertPfohl-Leszkowicz, AnnieAccinelli, CesareHallegraeff, Gustaaf M.Pietri, AmedeoAgeitos, JoseHallsworth, J.E.Pincus, SethAhmadi-Afzadi, MasoudHamilton, DavidPinotti, LucianoAhn, Tae SeokHammes, Mary S.Pinton, PhilippeAimanianda, Vishu KumarHan, Sang-MiPitt, JohnAKIO, NAKANEHans, GuyPizzolotto, RobertoAlagon, AlejandroHardy, MargaretPlaczek, RichardAlam, Mohammad ParvezHarrison, Lisa MPlaza-Zabala, AinhoaAlbrecht, Eric A.Harvey, AdamPollastro, StefaniaAlcaide, F. J. E.Hassan, MohamedPopov, SergueiAlexander, DenisHassan, Yousef I.Posselt, GernotAlgood, HollyHatano, TsutomuPourahmad, JalalAli, NurshadHautbergue, GuillaumePovey, AndrewAlloza, IraideHeit, BryanPREVOST, GillesAlonso, Juan CarlosHendrich, SuzanneProft, ThomasAltemimi, AmmarHermand, DamienPrusky, DovAlter, KatharineHerzig, VolkerPuddick, JonathanAlthaus, MikeHeuck, AlejandroPulido, DavidAlvito, Paula CristinaHeuck, Alejandro P.Punga, Anna RostedtAmar, LaurenceHildebrandt, Jan-PeterQuinton, LoïcAmetaj, BurimHiskia, AnastasiaRamachandran, SrinivasanAmiche, MohamedHo, MengfeiRamani, VijayAna, Juan-GarcíaHofmann, UteRamasamy, SrinivasanAnas, Andrea RoxanneHolmes, MarkRamirez, ManuelAndersson, Håkan SHook, IngridRasche, LeoAndjelkovic, MirjanaHopping, GeneRasmussen, Peter HaveAndrade, María J.Hoskins, ClareRassoulzadegan, MinooAndrianasolo, EricHosório, HugoRediske, Richard R.Andy Pickett, AndyHoward, Meredith D.a.Reinach, Peter S.Anfossi, LauraHsuan, J.Resiere, DaborAnglister, JacobHu, Kyung-SeokRestivo, Francesco MariaAnniballi, FabrizioHu, Wei-GangRestivo, DomenicoAppell, MichaelHuang, Tur-FuReuter, KlausArakawa, OsamuHuang, YadongReveillon, DamienAraoz, RómuloHuang, Guan-JhongReverberi, MassimoAraujo, PedroHuang, Xiao (Eric)Reyes, Victor E.Arbuckle, KevinHübner, FlorianRibet, DavidArbuckle, KevinHughes, ScottRiccardo, Zenezini ChiozziAriño, AgustínHughson, Michael D.Ricci, V.Armstrong, GlenHumpf, Hans-UlrichRicelli, AlessandraAthanassiou, ChristosHung, Dong-ZongRichardson, SaimonAung, Meiji SoeHungerford, JamesRitieni, AlbertoAuvynet, ConstanceHust, MichaelRizzuti, BrunoAviles, Francesc XavierHymery, NolwennRobert, ChristelleAwayda, MouhamedIadarola, PaoloRodgers, Kenneth J.Azarnia Tehran, DomenicoIaniri, GiuseppeRodríguez, AliciaBackert, SteffenIijima, NoriakiRodriguez De La Vega, RicardoBackert, SteffenInagaki, HidetoshiRodríguez-Carrasco, YelkoBailey, Andy M.Intiso, DomenicoRoelants, KimBailly, Jean-DenisInzana, ThomasRojo, Maria AngelesBanack, SandraIppoliti, RodolfoRolland, J.l.Bang, Moon SukIrrgang, K.-D.Röltgen, KatharinaBang, Woo YoungIsbister, Geoffrey K.Romeis, JoergBarman, ApurbaIvanova, BojidarkaRonit, MazorBarrientos Velazquez, Ana L.Iverson, Nicole M.Rosenberger, Thad A.Barrington, DaniJabbari, bahmanRosier, Peter F. W.Barthel, AndreaJackson Woo, Chit ShingRossi, FilippoBartolotti, MassimoJaiswal, RakeshRouse, RodneyBasak, Ajit K.Janmaat, AlidaRoutledge, MichaelBattaglia, EricJarvis, JodieRoze, Ludmila V.Battilani, PaolaJass, JanaRozhon, WilfriedBaxter, SimonJin, RongshengRubert, JosepBeaulieu, DeniseJin, ZhaoRuiu, LucaBeckel, J. M.Jobling, PhilRuiz De Escudero, InigoBelaganahalli, ManjunathaJohnson, Shannon L.Russo, PasqualeBentel, Jacqueline M.Johnson, Arlen W.Ryu, Ji-wonBenz, RolandJokela, JouniRzymski, PiotrBera, TapanJosenhans, ChristineSabatier, Jean-MarcBerger, MadeleineJoshi, BharatSachkova, MariaBermudez, MarcelJosic, DjuroSaikumar, PothanaBerrada, HoudaJuan, CristinaSaito, HideyukiBerry, John P.Kahlert, StefanSaito, YoshiroBertuzzi, TerenzioKajiwara, SusumuSakudo, AkikazuBeversdorf, LucasKalb, Suzanne R.Salud Bea, Roberto De LaBevilacqua, AntonioKalkum, MarkusSan Martin, CarmenBideshi, DennisKaloudis, TriantafyllosSandrini, GiovanniBideshi, DennisKaminskyj, Susan G.W.Sangaraju, DewakarBilliald, PhilippeKamitani, ShigekiSanny, Charles G.Blackwell, BarbaraKamm, ChristophSansone, ClementinaBlangy, AnneKammerer, Richard A.Santacroce, LuigiBlasi, JuanKankaanpaa, HarriSantamato, AndreaBöhm, JosefKarlovsky, PetrSantini, AntonelloBosso, LucianoKarlsson, IdaSantos-Gallego, CarlosBotana, Ana M.Karmakar, ParthaSanzani, Simona MariannaBotti, MaddalenaKarp, Barbara IllowskySauka, DiegoBouaïcha, NoureddineKataoka, YoskySavoie, Jean-MichelBougis, PierreKatayama, TsutomuSawa, TeijiBoundy, Michael J.Katikou, PanagiotaSberveglieri, VeronicaBourne, Christina R.Kellenberger, StephanScala, ValeriaBradley, Walter G.Keller, JuliaScala, ValeriaBrantl, SabineKem, William RSchaumburg, FriederBraun, VolkmarKim, JamesSchelin, JennyBreves, GerhardKim, KyounghyunSchirmer, BastianBrigotti, MaurizioKim, Hee-JinSchneider, BarbaraBrown, DarenKim, Sun KwangSchroeckh, VolkerBurguete, Gerardo CorzoKim, EuikyungSchroeder, CristinaCabral, CéliaKimura, TadashiSchrum, AdamCalleros, LauraKimura, HidetoSchuster, Christopher F.Callery, Patrick S.Kiris, ErkanSchwan, William R.Calvete, Juan J.Kitamura, NaokiSchweiger, WolfgangCâmara, JoséKJ, ClemetsonSchwerdt, GeraldCamarero, Julio A.Klassen, RoalndSella, LucaCaméan, AnaKlotz, JamesSerra, Pier AndreaCarmeli, ShmuelKluess, JeannetteServent, DenisCarreras, JoaquimKlüß, JeannetteSesardic, DorotheaCavaliere, ChiaraKnopp, DietmarSgouras, DionyssiosCegolon, LucaKobayashi, NobumichiSharma, RashmiCerasino, LeonardoKoch, MatthiasSharma, SushilChan, Y. K.Koelman, J. H. T. M.Shen, ZhenyuChan, LeeKolesárová, AdrianaShepherd, Suzanne M.Chanda, AnindyaKollewe, KatjaShewale, SwapnilChang, Perng KuangKoneru, BhuvaneswariShiea, JentaieChang, Chiou LingKonno, KatsuhiroShimada, YasuhitoChang, Long-SenKool, JeroenShinada, TetsuroChao, Louis KuopingKotta-Loizou, IolyShityakov, SergeyChase, ChristopherKouadio, James HalbinSidiropoulos, ChristosChen, JianwuKovbasnjuk, OlgaSilipo, AlbaChen, TianbaoKrähenbühl, StephanSilva, AnjanaChen, Chun-ChiKrepkiy, Dmitriy VSilva, Christopher J.Cheng, WaiwaiKretzschmar, Anna LizaSimmonds, RachelCheng, Ann-liiKrock, BerndSinger, Barbara J.Cheriyath, VenugopalanKubo, TaiSingh, ArunimaChevrot, RomainKudryashov, Dmitri S.Singh, Bal RamChiang, Tai-anKuntz, MarcelSiniscalco, DarioChijiwa, TakahitoKunze, GotthardSionov, EdwardChirumbolo, SalvatoreKurosawa, ShinichiroSirsat, SujataChitalia, VipulKushiro, MasayoSmith, Sinead M.Chitarra, WalterLadeira, CarinaSmith, G. JasonChizzola, RemigiusLai, RenSmith, JenniferChoi, Shin SikLam, Kwok HoSmout, MichaelChristina, SpryLaskin, DanielSoares, RaquelChu, XiangpingLattanzio, Veronica Maria TeresaSolfrizzo, MicheleChung, Soo HyunLausten, AndreasSondergaard, Teis EsbenChung, Man-KyoLee, Moo-SeungSørensen, Jens LauridsChyau, Charng-CherngLee, Hyang BurmSoulage, ChristopheCirés, SamuelLee, Chang HoonSousa, SandraCiriaco, FulvioLefebvre, David E.Soutourina, OlgaCirrincione, GirolamoLegros, ChristianSparks, DarrellCitores, LuciaLehman, P. W.Spooner, Robert AnthonyCohen, GeraldLeitão, AnaSreedasyam, AvinashColinet, DominiqueLemichez, EmanuelSrivenugopal, KalkunteComitini, FrancescaLemmens-Gruber, RosaStarace, MichelaComte, KatiaLevy, HaimStatsyuk, Natalia V.Conti, LuciaLi, YizhenStaunton, SiobhánCooke, Ira R.Li, Ying-JiStearns-Kurosawa, Deborah J.Cooke, MarcusLi, Mei-LingStępień, ŁukaszCordoba-Diaz, D.Lim, CheeStiles, BradleyCortinovis, CristinaLin, Hsu YangStirpe, FiorenzoCostantini, FrancescaLindkvist-Petersson, KarinStoll, DominicCotella, DiegoLing, ChenStubbs, EvanCover, TimothyLiu, ChengSturm, Gunter J.Cox, Russell J.Llewellyn, Lyndon E.Suarez, DimasCrickmore, NeilLoboda, AgnieszkaSubramaniam, RajagopalCristofori-Armstrong, BenLocatelli, FrancescoSugiura, KeijiCui, LongzhuLondon, ErwinSulyok, MichaelCunha, SaraLou, JianlongSun, ChangpoDall'Asta, ChiaraLouzao, M. CarmenSun, JianjunDaly, MegLu, WeiyueSunagar, KartikDaly, NorelleLuo, MengSundheim, LeifDänicke, SvenMa, YanSung, Wang ChouDaniels-Wells, Tracy R.Macrander, JasonSusca, AntoniaDart, Richard C.Macrander, JasonSuzuki, HodakaDashtipour, KhashaiarMahimainathan, LeninSwain, MartinDavid, Michael Z.Malliavin, ThérèseSwevers, LucDavid, Michael Z.Mancheño, JoséSycha, ThomasDavletov, BazbekManes, JordiTadahiro, SuzukiDavolos, DomenicoManganiello, GelsominaTahriri, MohammadrezaDe Bernard, MarinaMantzoukas, SpiridonTamagnini, PaulaDe Gelder, LeenManyes, LaraTamburin, StefanoDe Girolamo, AnnalisaMaragos, ChrisTartaglione, LucianaDe Haro, LucMaresca, MarcTatulian, Suren A.De La Cruz, Armah A.Margres, MarkTaylor, MatthewDe Petrocellis, LucianoMarin, DanielaTerradot, LaurentDe Spiegeleer, BartMarino, AngelaTerzi, ValeriaDegen, GiselaMariottini, Gian LuigiTerzi, ValeriaD'elios, Mario M.Markaki, PanagiotaTester, PatriciaD'Elios, Mario MilcoMarkland, FrankTeter, KenDellafiora, LucaMarte, AntonioTetreau, GuillaumeDelogu, GiovannaMartín, JuanThyagarajan, BaskaranDeo, RandhirMartinez-del-Pozo, AlvaroTietze, AlesiaDeplazes, EvelyneMartins, Ian JamesTirloni, EricaDeplazes, EvelyneMartinson, Ellen O.Tischler, DirkDerman, YagmurMaruyama, ToruTittlemier, Sheryl A.Dessain, ScottMarvin, ShaunaTolosa, JosefaDi Stefano, VitaMarzocco, StefaniaTomas, MariaDias, ElsaMatoba, NobuyukiTomaz, Cândida TeixeiraDiaz, DuarteMatsui, TaeiTonacci, AlessandroDidier, AndreaMatsumoto, TakuyaTortorella, DomenicoDiego-García, EliaMattei, CesarTouchard, AxelDimosthenis, KIZISMatten, Sharlene R.Toyotome, TakahitoDoekes, GertMaura, DamienTrachtman, HowardDomashevskiy, Artem V.Maynard, JenniferTremblay, Marie-LaurenceDong, MinMazor, OhadTrim, Steven A.Doona, ChristopherMcClain, Mark S.Tsuge, HideakiDorner, Martin B.McCormick, SusanTuchscherr, LorenaDossena, SilviaMcdermott, MichaelTudzynski, BettinaDoxey, AndrewMcdougal, Owen M.Tufféry, PierreDoyle, ThomasMcHenry, PeterTunaru, SorinDu Bois, JustinMcLaughlin, John E.Turrà, DavidDübel, StefanMebs, DietrichTzika, EleniDucancel, FrédéricMeca, GiuseppeUegaki, RyuichiDucatelle, RichardMegighian, AramUmemura, MycoDutertre, SebastienMeinhardt, FriedhelmUnger, Michael A.Easmon, JohnnyMelville, StephenUrra, José MiguelEhrich, MarionMetcalf, JamesVago, RiccardoEichacker, PeterMetcalf, Jordan P.Vale, CarmenEl Khalloufi, FatimaMeulenberg, ElineVale, PauloEllegaard, MarianneMeyer, Timothy W.Valentin-Weigand, PeterEl-Nezami, Hani SaidMeyer, Hans-PeterValério, ElisabeteEmani, ChandrakanthMézes, MiklósValiante, VitoEngmark, MikaelMicheli, LauraVan Coillie, ElsErny, Guillaume L.Michlmayr, HerbertVarga, ElisabethEspinosa, ManuelMiddeleer, Gilke DeVarga, ElisabethEsterhuizen-Londt, MarandaMikheyev, Alexander S.Vasconcelos, VítorEstevez, Jorge D.Miles, JohnVaughan, Martha MEtxebeste, OierMillar, John S.Vemula, HarikaFakhoury, AhmadMiller, DavidVenancio, ArmandoFalconer, IanMinervini, FiorenzaVenkatadri, RajkumarFalconer, IanMiyashita, MasahiroVettorazzi, ArianeFanelli, FrancescaMoar, William J.Viegas, SusanaFang, BaiMolot, Lewis A.Vignes, MichelFarrell, HazelMoon, YuseokVilcinskas, AndreasFasullo, MichaelMorita, KyojiVillablanca, EduardoFederici, BrianMoura, Ana MariaVillani, AlessandraFernandes, Ana S.Muench, StephenVisvikis, OraneFernandes, José OliveiraMukhopadhyay, SomshuvraVon Wright, AtteFernández-Cruz, María LuisaMunday, RexVoss, KennethFerreira, Rui M.Murch, SusanVrentas, Catherine E.Ferreras, José MiguelMurugesan, G. R.Wakino, ShuFerrero, RichardMusch, MarkWalker, AndrewFineran, PeterMüthing, JohannesWan, Lam YimFink-Gremmels, JohannaMutsaers, HenricusWang, YumengFinley, NatoshaNabok, AlexeiWang, YifeiFitzgerald, DavidNagashima, HitoshiWang, ZenengFlannery, BrennaNakamura, NorikoWang, ZeminFlavell, David J.Narracci, MarcellaWarriner, KeithFlorian, PitterlNarva, KennethWaterfield, NicholasFloriano, Wely B.Nathanail, AlexisWeaver, MarkFologea, DanielNaumann, MarkusWeber, BarbaraFont, GuillerminaNaumann, MichaelWebster, BenFox, Jay W.Nelsen, David R.Weenink, ErikFroeyen, MatheusNestor, CaseyWelch, Aaron Z.Frucht, David M.Nestorovich, EkaterinaWelgamage Don, AakashFruhmann, PhilippNg, KarlWeller, MichaelFuchs, HendrikNiculita-Hirzel, HélèneWeng, AlexanderFujinaga, YukakoNielsen, Vance G.Weng, AlexanderFusco, VincenzinaNiessner, ReinhardWheeler, DaveGajęcki, MaciejNoghero, AlessioWhyard, SteveGalanis, AlexNoguchi, TamaoWie, Myung-BokGalat, AndrzejNonami, AtsushiWięckiewicz, MieszkoGalaverna, GianniNtalli, Nikoletta G.Wilkenfeld, Ari Jacob LeviGall, BrianNunn, PeterWilliams, KatherineGallo, AntoniaOgashawara, IgorWilson, Brenda AnneGambino, G.O'Kennedy, RichardWong, Ching-OnGámiz-Gracia, LauraOkumura, ShiroWood, SusieGan, FeiOliver, RichardWoolbright, BenGarcia Ivars, JorgeOmur-Ozbek, PinarWozny, MaciejGarcía-Ortega, LucíaOrtiz-Urquiza, AlmudenaWu, FeliciaGarcía-Ortega, LucíaOscarsson, JanWürzner, ReinhardGarner, BiancaOshiro, NaomasaWuster, WolfgangGarrosa, ManuelOstolaza, HelenaWynendaele, EvelienGazerani, ParisaOtero, PazYamaguchi, YoshihiroGerardo, Charles JOtsuka, YuichiYao, GuoruiGerwin, RobertOtto, Angela M.Yao, JianxiuGhaste, ManojOyama, EtsukoYin, GuoweiGiannoni, PatriziaOzias-Akins, PeggyYli-Mattila, TapaniGiansanti, FrancescoPadula, AndrewYong, Tai-soonGilabert-Oriol, RogerPagliara, PatriziaYoshinari, TomoyaGilbert, Robert J.C.Pak, Sok CheonYu, Yan PingGillet, DanielPalanisamy, Satheesh KumarYu, HaiGilliam, Lyndi L.Palanisamy, Satheesh KumarYu, Jae-HyukGiovannoli, CristinaPalma, LeopoldoZamaratskaia, GaliaGirbes, TomasPalma, Mario SergioZeleny, ReinhardGkelis, SpyrosPalmieri, DarioZhang, KaiGnavi, GiorgioPanagou, EfstathiosZhang, FanGoldmana, Ellen R.Park, Hyun-WooZhao, RuimingGoldoni, MatteoPark, Kwan-KyuZhao, FeiGómez-Muñoz, AntonioPark, WansuZhbannikov, Ilya Y.Gonkowski, SlawomirParker, DaneZhou, Carol L. EcaleGóral, TomaszPascual, Samuel IgnacioZhu, FangGorrell, RebeccaPascual-Ahuir, AmparoZichel, RanGraham, H. KerrPasini, LuigiZilberberg, NoamGrant, GeorgePatel, Hardikkumarzimba, paulGreen, BenPathak, RupakZobel-Thropp, Pamela A.Greene, Lesley H.Peigneur, SteveZoupa, MariaGris, DenisPelin, MarcoZuliani, Juliana PavanGromadzka, KarolinaPellett, SabineZupo, ValerioGrubisha, LisaPereira, Ana LuisaZutz, ChristophGuerre, PhilippePérez-Pascual, DavidZygadlo, JulioGuillou, HervéPerrier, Josette

